# Four new species of the genus *Diduga* Moore, [1887] (Lepidoptera, Erebidae, Arctiinae) from China and Malaysia

**DOI:** 10.3897/zookeys.985.54047

**Published:** 2020-11-05

**Authors:** Ting Ting Zhao, Hui Lin Han

**Affiliations:** 1 School of Forestry, Northeast Forestry University, Harbin, 150040, China Northeast Forestry University Harbin China; 2 Key Laboratory of Sustainable Forest Ecosystem Management-Ministry of Education, Northeast Forestry University, Harbin, 150040, China Northeast Forestry University Harbin China

**Keywords:** Lithosiini, morphology, moth, Southeast Asia, taxonomy

## Abstract

In this paper, four species of the genus *Diduga* Moore, [1887] from China (Chongqing and Guangdong) and Malaysia (Borneo, Sabah) are described as new to science, namely *D. simianshana***sp. nov.**, *D. chebalinga***sp. nov.**, *D. chewi***sp. nov.**, and *D. hollowayi***sp. nov.** Adults of these species are illustrated in color, and images of the male and female genitalia are provided. A distribution map of the new species is provided, together with an updated checklist of all species of *Diduga*.

## Introduction

The genus *Diduga* belongs to the tribe Lithosiini in the subfamily Arctiinae, and was established by Moore ([1887], in 1884–1887), based on the type species *Diduga costata* Moore, [1887] from Dickoya, Sri Lanka. Before the establishment of the genus *Diduga*, Snellen (1879) had published a new species from India as *Pitane flavicostata*. Between 1891 and 1918, Hampson (1891, 1900, 1911, 1914, 1918) studied the genus *Diduga* and described eleven new species from the Oriental and Australian regions. After that, *D. haematomiformis* Eecke, 1920 was described from Indonesia.

Subsequently, the study of the genus entered a stage of stagnation until the turn of the new century. Fang (2000) recorded *D. flavicostata* from China. Holloway (2001) reviewed the faunistics and systematics of Bornean Lithosiini and recorded five species of *Diduga*, including three news ones, namely *D. barlowi*, *D. ciliata*, and *D. dorsolobata*. More recently, Černý and Pinratana (2009), Bucsek (2012, 2014), Singh et al. (2014), Bayarsaikhan et al. (2018, 2019, 2020), Bae et al. (2019), and Bucsek (2020) have described a total of 22 new species from Southeast Asia (see checklist). To date, this genus comprises therefore 39 described species worldwide, with the majority (25) described in the past two decades.

## Materials and methods

The specimens were collected using a 220V/450W mercury light and a DC black light in Chongqing Municipality (Mt. Simian), Guangdong Province (Chebaling), China, and the Borneo Jungle Girl Camp, Malaysia. Standard methods for dissection and preparation of genitalia slides were followed Kononenko and Han (2007). The vesicae were not everted and the relative position of cornuti along them is given as if they had been everted. Specimens were photographed using a Nikon D700 camera; the genitalia slides were photographed using an Olympus photo microscope controlled via Helicon Focus software, further processed in Adobe Photoshop CS6. The type materials of the new taxa are deposited in the collection of Northeast Forestry University, Harbin, China.

Abbreviations used:

**NEFU** Northeast Forestry University, Harbin, China

**TL** Type locality

**TS** Type species

## Taxonomic account

### Family Erebidae Leach, [1815]

**Subfamily Arctiinae Leach, [1815**]

### Tribe Lithosiini Billberg, 1820

#### Genus Diduga

Taxon classificationAnimaliaLepidopteraArctiidae

Moore, [1887]

88E827B2-9163-5805-B765-934B6B4CA66F

Diduga Moore, [1887]. The Lepidoptera of Ceylon 3 (4): 535. TS: Diduga costata Moore, [1887]. TL: Ceylon, [= Sri Lanka], Dickoya.
= Androstigma Hampson, 1893. Illustrations of typical specimens of LepidopteraHeterocera in the collection of the British Museum 9: 13, 82. TS: Diduga albicosta Hampson, 1891. TL: India, Nilgiri Plateau.

##### Diagnosis.

Species of *Diduga* are small in size. The proboscis is fully developed, the labial palpus is slender, directed upwards over the top of the head; the male antennae vary from ciliated to bipectinated. The tibial spurs are long.

In the male abdomen, the 8^th^ tergite is narrowed, with long and slender apodemes (Fig. 13); and the genitalia has narrow but long lateral hairpencils in many species. The configuration of valva may vary; usually they are simple, long, slender, and tapered, or short with several distinct processes, sometimes showing bilateral asymmetry. In the female genitalia, the ductus and corpus bursae range considerably in length.

Most species have brown forewings with white or yellowish margins along the costa and distally, or forewings are medium brown with darker fasciae and stigmata. There are often various hairpencils and androconial tufts on the wings of males. In addition, the forewings have a complete set of veins arising from the cell, R_1_ to R_3_ all extending to the costal margin, R_4_ and R_5_ have a common stem, the others are independent. In the hindwing, R_S_ and M_1_ have a common stem, M_2_ is absent, the others are independent (Fang 2000; Holloway 2001).

#### Diduga simianshana sp. nov.

Taxon classificationAnimaliaLepidopteraArctiidae

2A11A770-EFC5-547B-9B94-45DE77B9593F

http://zoobank.org/2598C94C-A258-4CEE-96EC-6FA25FF15A06

**Figures 1–6.**
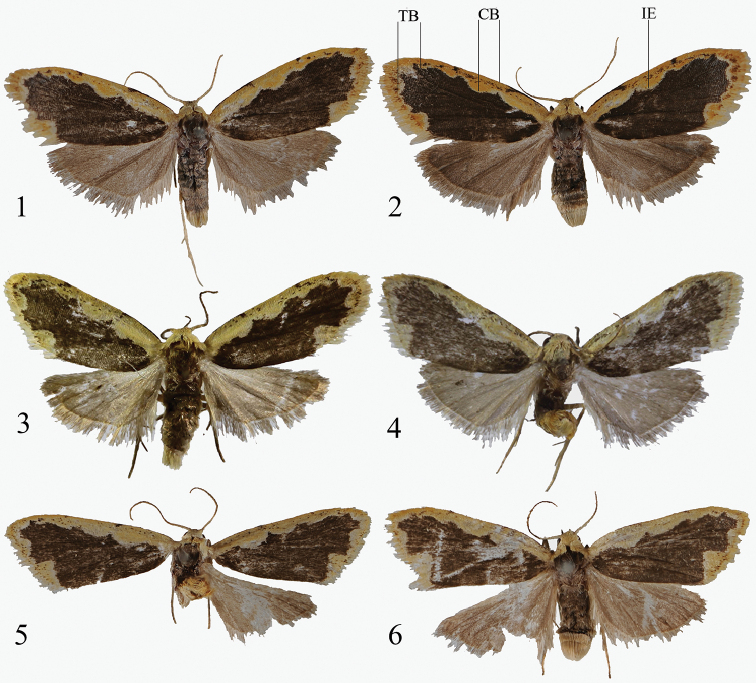
Adults of *Diduga* spp. **1***D. simianshana* sp. nov., male, holotype, China (Chongqing) **2** dittos, female, paratype, China (Chongqing) **3***D. nigridentata*, male (after Bayarsaikhan and Bae 2019) **4***D. quinquicornuta*, male (after Bayarsaikhan and Bae 2019) **5***D. chebalinga* sp. nov., male, holotype, China (Guangdong) **6** ditto, female, paratype, China (Guangdong). CB: costal band; IE: inner edge of costal band; TB: terminal band.

Figs 1 , 2 , 14 , 22 , 28

##### Material examined.

***Holotype*:** China: ♂; Chongqing, Jiangjin, Mt. Simian; 28.584°N, 106.356°E, elevation 1103 m; 12–13.VII.2018; leg. H.L. Han & C. Zhang; genit. prep. no. ztt-070-1; in NEFU. ***Paratypes*:** 3♀♀; same data as holotype; genit. prep. nos. ztt-073-2, ztt-074-2, ztt-080-2; in NEFU.

##### Diagnosis.

The new species is externally similar to *D. nigridentata* Bayarsaikhan & Bae, 2019 (Figs 3, 15). It can be separated from the latter by the following characters (*D. nigridentata* details are between parentheses): the ground color of the forewing is darker; an approximate right-angled bulge at tornal area (with arched bulge); the ground color of the hindwing is darker, in the male genitalia, the two basal projections of valva are longer than tegumen (shorter); the left cucullus bears two small spines (only a single long spine); the right cucullus and costal process are fused (separated); the cornutus is long and straight (short and arched).

##### Description.

***Adult*:** (Figs 1, 2) Wingspan 13–14 mm. Head yellow; antenna filiform, brown. Thorax dark brown, patagium and tegula yellow. Abdomen with pale yellow anal tuft. Forewing with dark brown ground color; costal band broad, yellow, with several small, dark brown dots, its inner edge undulated; median line absents at costa but present as a dot at the inside inner edge of costal band; terminal band of same color as costal one, inner edge of terminal band undulated, with an approximate right-angled bulge at tornus; terminal line with an admixture of brown and yellow; fringe yellow. Hindwing smoky brown, with diffuse, small, dark brown flecks; fringe pale to smoky brown, light yellow basally. ***Male genitalia*** (Fig. 14). Tegumen triangular, weakly sclerotized, as long as uncus; the basal projections of tegumen asymmetrical, strongly sclerotized, clavate, with a triangular denticle distally; left one with large hemispherical tubercle distally, right one fist-sharped. Vinculum U-shaped, sclerotized, thick. Valva rather flat, broad, asymmetrical; the left one longer and wider; sacculus long, mostly straight, barely shorter than whole valva, saccular process curved, fingerlike, with long, sclerotized terminal spine; costa very narrow, thick, as long as valva; cucullus strongly sclerotized and tapered into elongate subtriangular process, with a long stout spine distally; the right one flat, sacculus narrow and straight, saccular process curved, fingerlike, with a shorter terminal spine; costa narrow; cucullus strongly sclerotized and tapered, with a long stout spines distally. Uncus sinuous, short and thick basally; swollen before pointed, hooked apex. Aedeagus cylindrical, smoothly curved, coecum swollen and short, ca 1/9 as long as overall aedeagus; vesica with a small triangular cornutus, and a slender, long cornutus accompanied by a small band of hair. ***Female genitalia*** (Fig. 22). Ostium bursae rough and weakly sclerotized, the 8^th^ abdominal segment shaping a deeply invaginated V-shaped ostium bursae; two triangular lobes at both sides. The posterior margin of the 7^th^ abdominal has a distinctly sunken fold. The 6^th^ abdominal segment slightly thickened posteriorly, with slight ridges on both sides. Ductus bursae long and narrow, twisted at middle, its posterior half strongly sclerotized, flat and straight, and anterior one membranous, partly rugose. Corpus bursae globular, membranous, with a ring-shaped signum band. Apophysis anterior rather thick, ca 5/8 as long as apophysis posterior, this slender and long. Papillae anales cone-shaped, covered with setae.

##### Etymology.

The species is named after the type locality, Mt. Simian, Chongqing Municipality, China.

##### Distribution.

China (Chongqing: Mt. Simian) (Fig. 28).

##### Habitat.

The species was collected by light trap close to an evergreen broadleaf forest. The main tree species in the collecting biotope are *Engelhardia roxburghiana* Wall.,1831 and *Cunninghamia lanceolata* (Lambert) Hooker, 1827.

#### Diduga chebalinga sp. nov.

Taxon classificationAnimaliaLepidopteraArctiidae

158AEAA3-F102-5B73-B9CD-32C7CC7F3F19

http://zoobank.org/43F89C07-B094-454F-946B-85BAF7C9DA71

Figs 5 , 6 , 16 , 23 , 28

##### Material examined.

***Holotype*:** China: ♂, Prov. Guangdong, Shaoguan, Chebaling National Nature Reserve; 24.731°N, 114.267°E, elevation 463 m; 29.IV–3.V.2019; leg. H. L. Han & J. Wu; genit. prep. no. ztt-078-1; in NEFU. ***Paratype*:** 1♀; same data as holotype; genit. prep. no. ztt-077-2; in NEFU.

##### Diagnosis.

The new species is externally similar to *D. quinquicornuta* Bayarsaikhan & Bae, 2019 (Figs 4, 17, 24). It can be separated from the latter by the following characters (*D. quinquicornuta* details between parentheses): the wingspan is broader; the tegumen is thin (thick); the right costal process is long, wide, flat, and rounded distally (short, hornlike, sharp distally); the cucullus is sclerotized, thick, spoon-shaped, with a short horn distally (poorly sclerotized, club-shaped, wrinkled); the uncus is slightly swollen medially, not bending ventrally (wider and flat, hooked apex); in the female genitalia, the ostium bursae is strongly sclerotized, wrinkled, and bending to the left (weakly wrinkled, typical); the ductus bursae is curved, gradually broadening from anterior to posterior (typical, tapered); the corpus is divided into two parts, a posterior part membranous, and the anterior one globular, densely covered small flecks (long oval, anterior half with dense, small flecks, and more than 6 signa forming a vertical semicircle, posterior half membranous, smooth).

##### Description.

***Adult*:** (Figs 5, 6) Wingspan 13–14 mm, female larger than male. Head yellow; antenna filiform. Thorax dark brown; patagium, and tegula yellow. Abdomen brown, with pale yellow anal tuft. Forewing with dark brown ground color; costal band broad, yellow, with dispersed small, dark brown scales; its inner edge undulated; terminal band of same color as costal one, and its inner edge undulated, with an approximately right-angled bend near tornus; terminal line with an admixture of brown and yellow; fringe yellow. Hindwing smoky brown, fringe pale to smoky brown. ***Male genitalia*** (Fig. 16). Tegumen triangular, thin, and narrow superiorly. Vinculum broadly V-shaped, sclerotized, thick. Valva asymmetrical and bifurcated; in the left one, basal projections of valva bifurcated, one short, the other one ca 6 times as long as the short one; sacculus thick, gradually broadening distally; saccular process narrow, smoothly arched; costa rather broad, smooth, with a curved, cone-shaped ampulla; costal process strongly sclerotized, thick, wedge-shaped; harpe short, cone-shaped, strongly sclerotized, cucullus spoon-shaped, with short, sclerotized horns distally; in the right one, sacculus weakly sclerotized, swollen, basal process lumpy; saccular process strongly sclerotized, short, sharp distally; costa broad, smooth, with a long, flat ampulla; costal process membranose; cucullus strongly sclerotized, bifurcated, one slender, rounded distally, the other one finger-shaped, distally. Uncus thick, covered with setae, slightly swollen medially, ca 7/9 as long as tegumen. Aedeagus weakly sclerotized, with small bulge at coecum; vesica with a small flecks plate at ventral part of basal. ***Female genitalia*** (Fig. 23). Ostium bursae broad, bent to left, with thick and sclerotized frame; lamella antevaginalis tongue-shaped. The 7^th^ abdomere strongly sclerotized, wrinkled, densely covered with setae. Ductus bursae curved, gradually broadening posteriorly. Corpus bursae divided, anterior half globular, densely covered with small flecks, posterior half membranous, thin, and wrinkled. Papillae anales broad, covered with setae.

##### Etymology.

The species is named after the type locality, Chebaling National Nature Reserve, Guangdong Province, China.

##### Distribution.

China (Guangdong: Chebaling) (Fig. 28).

##### Habitat.

The species was collected using a light trap close to a typical evergreen broadleaf forest of the mid-subtropics near the Zhangdong River. The main tree species in the collecting biotope is *Cunninghamia lanceolata* (Lambert) Hooker, 1827.

#### Diduga chewi sp. nov.

Taxon classificationAnimaliaLepidopteraArctiidae

A3EDA98A-BDE2-5A94-8C95-75CA6952B3EA

http://zoobank.org/74D5E4AE-3010-48D8-B167-FC83D367107

**Figures 7–12.**
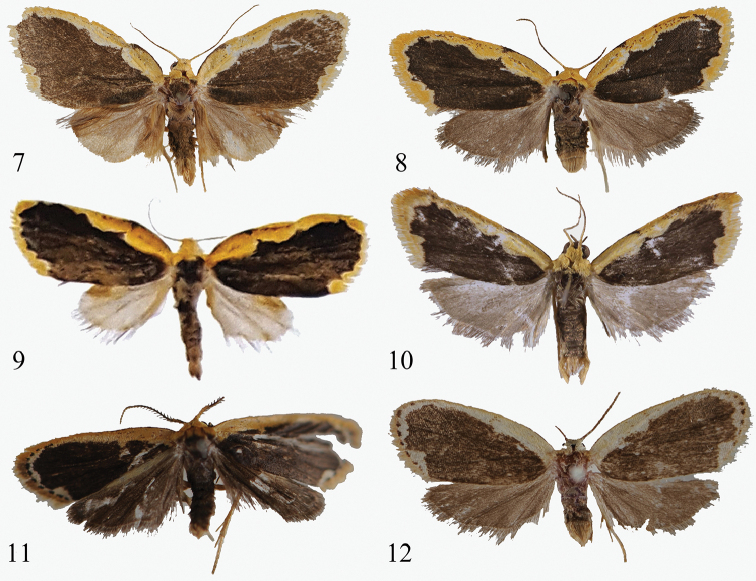
Adults of *Diduga* spp. **7***D. chewi* sp. nov., male, holotype, Malaysia (Borneo) **8** ditto, female, paratype, Malaysia (Borneo) **9***D. trichophora*, male (after Bucsek 2012) **10***D. kohkongensis*, male (after Bayarsaikhan & Bae, 2018) **11***D. hollowayi* sp. nov., male, holotype, Malaysia (Borneo) **12** ditto, female, paratype, Malaysia (Borneo).

Figs 7 , 8 , 18 , 25 , 28

##### Material examined.

***Holotype*:** Malaysia: ♂, Sabah, Borneo Jungle Girl Camp; 5.442°N, 116.451°E, elevation 1223 m; 15–20.II.2019; leg. H. L. Han; genit. prep. no. ztt-110-1; in NEFU. ***Paratypes*:** 1♂, 2♀♀; same data as holotype; genit. prep. nos. ztt-100-1, ztt-102-2, ztt-040-2; in NEFU.

**Figure 13.**
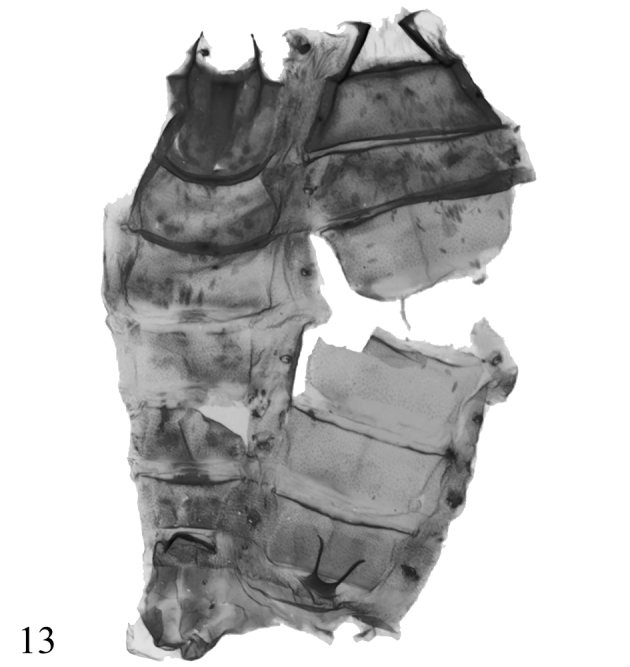
Abdomen of male adult of the genus *Diduga*.

##### Diagnosis.

The wing pattern of the new species is similar to that of *D. trichophora* Hampson, 1900 (Figs 9, 19). It can be separated from the latter by the following characters (*D. trichophora* details are between parentheses): the forewing is broader (narrow); the male hindwing is dark grey, broad fan-shaped (pale, narrow fan-shaped); in the male genitalia, the valva termination bifurcated distally (finger-shaped, sharp distally); the coecum is typical (bifurcated); the vesica has long, narrow band of flecks (without).

**Figures 14–17.**
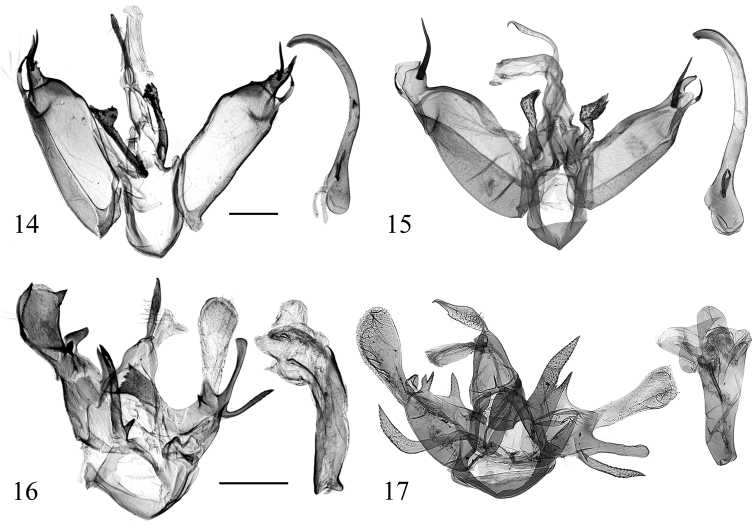
Male genitalia of *Diduga* spp. **14***D. simianshana* sp. nov., holotype, genit. prep. No. ztt-070-1 **15***D. nigridentata*, 2019 (after Bayarsaikhan and Bae 2019) **16***D. chebalinga* sp. nov., holotype, genit. prep. No. ztt-078-1 **17***D. quinquicornuta* (after Bayarsaikhan and Bae 2019). Scale bars: 0.5 mm.

##### Description.

**Figures 18–21.**
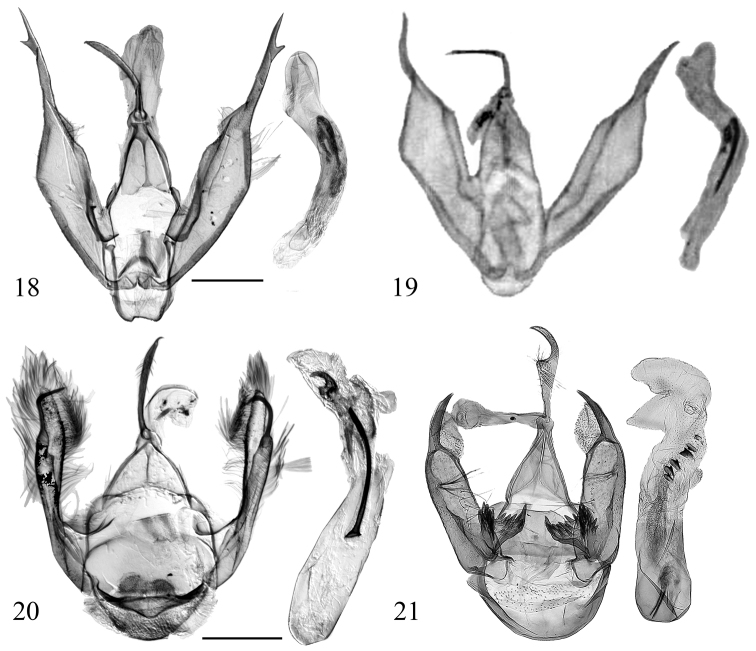
Male genitalia of *Diduga* spp. **18***D. chewi* sp. nov., holotype, genit. prep. No. ztt-110-1 **19***D. trichophora* (after Bucsek 2012) **20***D. hollowayi* sp. nov., holotype, genit. prep. No. ztt-033-1 **21***D. kohkongensis* (after Bayarsaikhan & Bae, 2018). Scale bars: 0.5 mm.

Adult (Figs 7, 8). Wingspan 15.5–16.5 mm. Head yellow; antenna filiform. Thorax dark brown; patagium, and tegula yellow. Abdomen dark to brown, the latter with pale yellow anal tuft. Forewing with dark brown ground color; veins and inner margin more black; costa slightly angled at 1/4 of the wing; costal band broad, yellow, with several dots and patches; its inner edge undulated; terminal band of same color as costal one, with internally facing concavity at tornus, the inner edge of terminal band undulated; terminal line and fringe yellow; ventral side of inner margin with long, brown hair tuft. Hindwing dark grey to smoky brown; costa with longer scales in male; tornus area sunken; fringe brown. ***Male genitalia*** (Fig. 18). Tegumen triangular, weakly sclerotized, slightly longer than uncus. Vinculum U-shaped, sclerotized. Juxta large, linguliform, weakly sclerotized, inverted harpoon-shaped. Valva approximately diamond-shaped, rather flat and symmetrical; sacculus narrow, thick and straight, 2/3 as long as valva, this terminated by long straight, sharply pointed process slightly bifurcated before apex. Uncus hooked and slender, sharp distally. Aedeagus curved, short, thick; caecum slightly swollen, ca 1/4 as long as whole aedeagus; vesica with a long cornutus and a scobinate band medially. ***Female genitalia*** (Fig. 25). Ostium bursae infundibuliform, weakly sclerotized. Ductus bursae narrow, flat, moderately sclerotized, sinuous anteriorly. Corpus bursae globular, membranous, with a ring-shaped signum band covered by small spines and flecks. Base of apophysis anterior is a long, inverted triangle; apophysis posterior slender, long, slightly longer than apophysis anterior; Papillae anales cylindrical, weakly sclerotized, covered with setae.

##### Etymology.

The name “*chewi*” refers to Mr J. Chew, who is a person in charge in the camp site where the species was collected.

##### Distribution.

Malaysia (Borneo: Sabah) (Fig. 28).

##### Habitat.

The species was collected in a tropical rain forest area. Podocarpaceae and Myrtaceae are richest families in the collecting biotope, and mosses such as *Himantocladium plumula* (Nees) Fleisch., 1908, *Hypopterygium tamarisci* Bridel ex C.Müller, 1850, *Fissidens wichurae* Broth. & Fleisch., 1899 are also abundant.

#### Diduga hollowayi sp. nov.

Taxon classificationAnimaliaLepidopteraArctiidae

53E528A4-B185-5DC3-BD6C-D7EAAA496028

http://zoobank.org/82C24CC3-2001-4479-B813-E3997A45C615

Figs 11 , 12 , 20 , 26 , 28

##### Material examined.

***Holotype*:** Malaysia: ♂, Sabah, Borneo Jungle Girl Camp; 5.442°N, 116.451°E, elevation 1123 m; 15–20.II.2019; leg. H. L. Han; genit. prep. no. ztt-033-1; in NEFU. ***Paratypes*:** 1♂; same locality as holotype; 24.IV–2.V.2016; leg. H. L. Han; genit. prep. no. ztt-085-1; 7♀♀; same data as holotype; leg. H. L. Han; genit. prep. nos. ztt-034-2, ztt-083-2, ztt-096-2, ztt-097-2, ztt-099-2, ztt-103-2, ztt-104-2; in NEFU.

##### Diagnosis.

The new species is similar to *D. kohkongensis* Bayarsaikhan & Bae, 2018 (Figs 10, 21, 27) but it can be separated from the latter by the following characters (*D. kohkongensis* details are between parentheses): the ground color of forewing is darker; the male antenna is bipectinate (ciliate); the inner edge of costal band approximately straight (undulate); the terminal line distinct, formed by brown dots (yellow); the ground color of the hindwing dark brown (grey); in the male genitalia, the editum is a small band, slightly bulging (formed by stout spines); the valva is narrow and asymmetrical (symmetrical, stout); the apical process of valva is slender, long spine-shaped, incurved inward terminally (strongly horn-shaped, weakly arched); the uncus is slender and hooked (with angular bulge ventrally); the vesica has two cornuti, one small, claw-shaped, the other long, slender, smoothly arched (a row of six irregular cornuti); in the female genitalia, the ductus bursae is narrower; the corpus bursae is approximately triangular, with a triangular signum band posteriorly (rectangular, membranous, with plate of small spines at anterior half).

**Figures 22–27.**
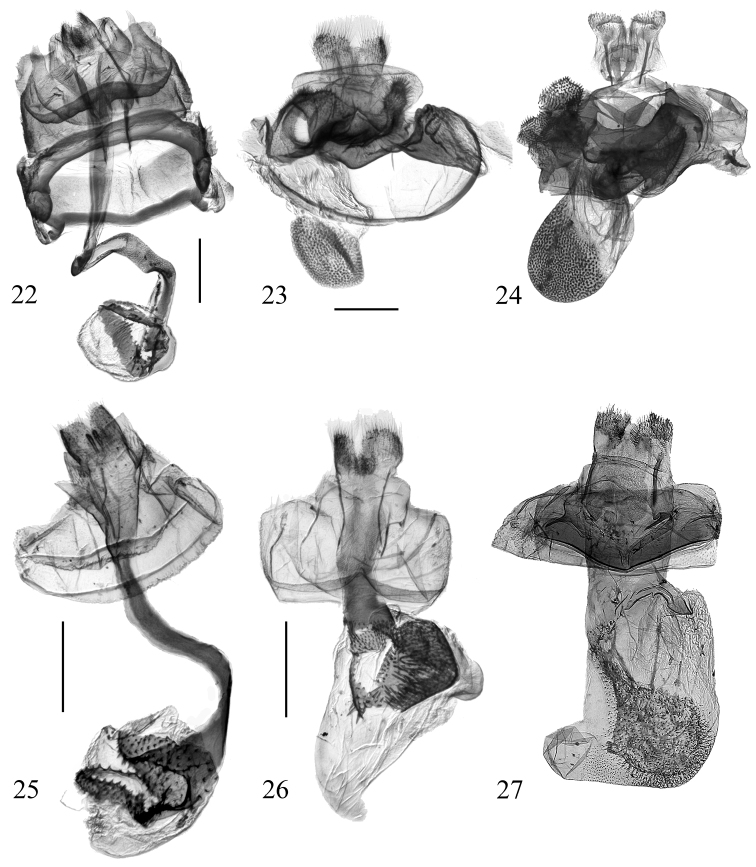
Female genitalia of *Diduga* spp. **22***D. simianshana* sp. nov., paratype, genit. prep. No. ztt-073-2 **23***D. chebalinga* sp. nov., paratype, genit. prep. No. ztt-077-2 **24***D. quinquicornuta* (after Bayarsaikhan and Bae 2019) **25***D. chewi* sp. nov., paratype, genit. prep. No. ztt-102-2 **26***D. hollowayi* sp. nov., paratype, genit. prep. No. ztt-083-2 **27***D. kohkongensis* (after Bayarsaikhan & Bae, 2018). Scale bars: 0.5 mm.

##### Description.

Adult (Figs 11, 12). Wingspan 12–13 mm, female larger than male. Head yellow; male antenna bipectinate, female antenna filiform. Thorax brown; patagium and tegula yellow, the color of female lighter. Abdomen with pale yellow anal tuft. Forewing with dark brown ground color; costal band broad, yellow to canary yellow, its inner edge nearly straight; the inner edge of terminal band undulated, with a slight right angle at tornus; terminal line conspicuous, formed by brown dots; fringe yellow. Hindwing brown, costal band light brown; fringe brown to smoky brown. ***Male genitalia*** (Fig. 20). Tegumen triangular, weakly sclerotized, as long as uncus. Vinculum narrow, weakly sclerotized, very broadly U-shaped, with slightly produced semicircular saccus. Juxta flat, moderately sclerotized. Valva band-shaped, weakly sclerotized, covered with setae, asymmetrical; left valva with broad and moderately sclerotized sacculus, 3/4 as long as overall valva, saccular process in shape of a long spine bent internally at ca 90°; right valva as long as left one, its saccular process like left one albeit evenly hooked internally; costa very narrow, as long as valva. Uncus slender, slightly hooked. Aedeagus membranous, cylindrical; coecum short, 1/5 as long as overall aedeagus; vesica with a small claw-shaped cornutus, and long, slender, smoothly arched cornutus subterminally. ***Female genitalia*** (Fig. 26). Ostium bursae flat and membranous. Ductus bursae flat, weakly sclerotized. Corpus bursae membranous, with a triangular signum band posteriorly; right part strongly sclerotized, with a signum plate covered long spines, terminally connected to ductus bursae. Apophysis anterior short, apophysis posterior ca 2 times as long as apophysis posterior. Papillae anales cylindrical, weakly sclerotized, covered with setae.

##### Etymology.

The species is named after Dr J.D. Holloway, who conducted outstanding lepidopterological research in Borneo.

##### Distribution.

**Figure 28.**
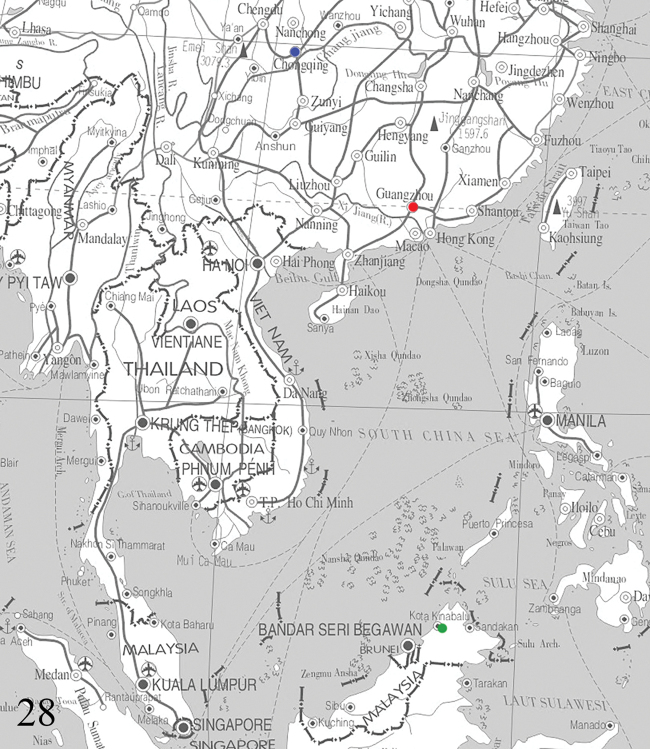
Collecting sites of *Diduga* spp. Key: *D. simianshana* sp. nov.: China, Chongqing, Mt Simian (blue dot); *D. chebalinga* sp. nov.: China, Prov. Guangdong, Shaoguan, Chebaling (red dot); *D. chewi* sp. nov. and *D. hollowayi* sp. nov.: Malaysia, Borneo (both green dot).

Malaysia (Borneo: Sabah) (Fig. 28).

##### Habitat.

The species was collected in a tropical rain forest area. Podocarpaceae and Myrtaceae are richest families in the collecting biotope, and mosses of *Himantocladium plumula* (Nees) Fleisch., 1908, *Hypopterygium tamarisci* Bridel ex C.Müller, 1850, *Fissidens wichurae* Broth. & Fleisch., 1899 are also abundant.

### Checklist of species in the genus *Diduga* Moore, [1887], with type localities

*Diduga albicosta* Hampson, 1891 (India: Nilgiris)

*Diduga albida* Hampson, 1914 (New Guinea: Mimika River)

*Diduga allodubatolovi* Bayarsaikhan, Li & Bae, 2020 (China: Yunnan)

*Diduga alternota* Bucsek, 2014 (Malaysia: Pahang)

*Diduga ambigua* Bucsek, 2014 (Malaysia: Perak)

*Diduga amoenusa* Bucsek, 2012 (Malaysia: Pahang)

*Diduga annulata* Hampson, 1900 (Indonesia: Sambawa)

*Diduga barlowi* Holloway, 2001 (Borneo: Brunei)

*Diduga bayartogtokhi* Bayarsaikhan & Bae, 2019 (Vietnam: Vinh Phuc)

*Diduga bispinosa* Bayarsaikhan & Bae, 2018 (Cambodia: Koh Kong)

*Diduga chebalinga* sp. nov. (China: Guangdong)

*Diduga chewi* sp. nov. (Malaysia [Borneo]: Sabah)

*Diduga ciliata* Holloway, 2001 (Borneo: Pulo Laut)

*Diduga costata* Moore, [1887] (Sri Lanka: Dickoya)

*Diduga cucphuonga* Dubatolov & Bucsek, 2016 (North Vietnam: Ninh Binh)

*Diduga dorsolobata* Holloway, 2001 (Borneo: Mt. Kinabalu)

*Diduga dubatolovi* Bayarsaikhan & Bae, 2018 (Cambodia: Koh Kong)

*Diduga excisa* Hampson, 1918 (Philippines: Luzon)

*Diduga flavicostata* (Snellen, 1879) (India: Nilgiris)

*Diduga flavifinis* Bucsek, 2014 (Malaysia: Perak)

*Diduga fumipennis* Hampson, 1891 (India: Nilgiris)

*Diduga khounngeuna* Bucsek, 2020 (Laos: Ban Khoun Ngeun)

*Diduga haematomiformis* van Eecke, 1920 (Indonesia: West Java)

*Diduga hainanensis* Bayarsaikhan, Li & Bae, 2020 (China: Hainan)

*Diduga hanoiensis* Bayarsaikhan & Bae, 2019 (Vietnam: Hanoi)

*Diduga hollowayi* sp. nov. (Malaysia [Borneo]: Sabah)

*Diduga iriomotensis* Bae, Kishida & Bayarsaikhan, 2019 (Japan: Okinawa)

*Diduga kohkongensis* Bayarsaikhan & Bae, 2018 (Cambodia: Koh Kong)

*Diduga luteogibbosa* Bayarsaikhan, Li & Bae, 2020 (China: Yunnan)

*Diduga macroplaga* (Hampson, 1900) (Indonesia [Borneo]: Pulo Laut)

*Diduga metaleuca* Hampson, 1918 (Philippines: Luzon)

*Diduga mininota* Bucsek, 2014 (Malaysia: Negeri Sembilan)

*Diduga nigridentata* Bayarsaikhan & Bae, 2019 (Vietnam: Hanoi)

*Diduga nota* Bucsek, 2012 (Malaysia: Pahang)

*Diduga pectinifer* Hampson, 1900 (Indonesia [Borneo]: Pulo Laut)

*Diduga plumosa* Hampson, 1911 (Indonesia: Sambawa)

*Diduga quinquicornuta* Bayarsaikhan & Bae, 2019 (Vietnam: Hanoi)

*Diduga rufidisca* Hampson, 1898 (India: Assam)

*Diduga scalprata* Bayarsaikhan, Li & Bae, 2020 (China: Yunnan)

*Diduga simianshana* sp. nov. (China: Chongqing)

*Diduga spinosusa* Bucsek, 2012 (Malaysia: Perak)

*Diduga trichophora* Hampson, 1900 (Indonesia [Borneo]: Pulo Laut)

*Diduga zetes* Bucsek, 2014 (Malaysia: Perak)

## Supplementary Material

XML Treatment for Diduga

XML Treatment for Diduga simianshana

XML Treatment for Diduga chebalinga

XML Treatment for Diduga chewi

XML Treatment for Diduga hollowayi
